# B-Type Natriuretic Peptide-Induced Delayed Modulation of TRPV1 and P2X3 Receptors of Mouse Trigeminal Sensory Neurons

**DOI:** 10.1371/journal.pone.0081138

**Published:** 2013-11-27

**Authors:** Sandra Vilotti, Anna Marchenkova, Niels Ntamati, Andrea Nistri

**Affiliations:** Neuroscience Department, International School for Advanced Studies (SISSA), Trieste, Italy; Dalhousie University, Canada

## Abstract

Important pain transducers of noxious stimuli are small- and medium-diameter sensory neurons that express transient receptor vanilloid-1 (TRPV1) channels and/or adenosine triphosphate (ATP)-gated P2X3 receptors whose activity is upregulated by endogenous neuropeptides in acute and chronic pain models. Little is known about the role of endogenous modulators in restraining the expression and function of TRPV1 and P2X3 receptors. In dorsal root ganglia, evidence supports the involvement of the natriuretic peptide system in the modulation of nociceptive transmission especially via the B-type natriuretic peptide (BNP) that activates the natriuretic peptide receptor-A (NPR-A) to downregulate sensory neuron excitability. Since the role of BNP in trigeminal ganglia (TG) is unclear, we investigated the expression of BNP in mouse TG in situ or in primary cultures and its effect on P2X3 and TRPV1 receptors of patch-clamped cultured neurons. Against scant expression of BNP, almost all neurons expressed NPR-A at membrane level. While BNP rapidly increased cGMP production and Akt kinase phosphorylation, there was no early change in passive neuronal properties or responses to capsaicin, α,β-meATP or GABA. Nonetheless, 24 h application of BNP depressed TRPV1 mediated currents (an effect blocked by the NPR-A antagonist anantin) without changing responses to α,β-meATP or GABA. Anantin alone decreased basal cGMP production and enhanced control α,β-meATP-evoked responses, implying constitutive regulation of P2X3 receptors by ambient BNP. These data suggest a slow modulatory action by BNP on TRPV1 and P2X3 receptors outlining the role of this peptide as a negative regulator of trigeminal sensory neuron excitability to nociceptive stimuli.

## Introduction

Sensory inputs, including painful and tissue-damaging stimuli, are conveyed from the periphery to the central nervous system through primary afferent neurons located in the trigeminal ganglia (TG) and dorsal root ganglia (DRG). Small- and medium-diameter sensory neurons (nociceptors) express, amongst a range of membrane proteins detecting noxious stimuli, capsaicin (and heat)-sensitive transient receptor potential vanilloid-1 (TRPV1) channels and/or adenosine triphosphate (ATP)-gated P2X3 subunit-containing receptors [1,2] to transduce pain. In particular, several studies have demonstrated TRPV1 to be essential for the development of inflammatory thermal pain conditions [3–5]. Among ATP-gated P2X receptors, the P2X3 receptors are almost exclusively expressed by sensory ganglion neurons [6,7] and have been implicated in craniofacial pain [8,9], including migraine [10]. The activity of TRPV1 and P2X3 receptors is known to be upregulated by endogenous peptides, like bradykinin, CGRP or substance P [11–15], and trophic factors like NGF and BDNF [16–18]. Thus, the functional action of such modulators is manifested as sensitization of these receptors, thereby contributing to lowering pain threshold and to triggering pain, especially of chronic nature. In this sense, their role on trigeminal sensory neurons as facilitators of migraine pain has been proposed [14,19–21]. As recently reviewed [22,23], clinical studies have confirmed that P2X3 and TRPV1 receptors mediate pain induced by distinct stimuli in man. 

Less is known about the potential role of endogenous modulators in restraining the expression and function of TRPV1 and P2X3 receptors. Recent evidence supports a potential involvement of the natriuretic peptide system in the modulation of sensory neuron nociceptive transmission [24–27]. Natriuretic peptides (NPs) are a family of structurally related paracrine factors, namely atrial NP (ANP), B-type NP (BNP), also known as brain natriuretic peptide, and C-type NP (CNP) [28]. ANP administration does not affect sensitivity to radiant heat [29] or mechanical allodynia [26,27], while CNP is proposed as a positive modulator of chronic pain [24]. Conversely, microarray gene profiling has indicated that chronic pain enhances BNP and its natriuretic peptide receptor-A (NPR-A) in rat DRG. Moreover, BNP application reduces the excitability of DRG nociceptors and the hyperalgesic response in a rat model of inflammatory pain. This led to the suggestion that BNP may play an inhibitory role in chronic pain [25]. BNP acts through binding to NPR-A, which is a guanylyl cyclase receptor (also sensitive to ANP), and increases intracellular cGMP levels [30,31].

While all NPRs have been identified in brainstem trigeminal nuclei [32–34], little is known about the possible role of the natriuretic peptide system at TG level where nociceptive signals are transduced [35–37]. A recent clinical report has shown that BNP levels are raised in the jugular vein blood during a migraine attack [38]. We have developed an in vitro model system using primary cultures of mouse TG to investigate the cellular mechanisms regulating the expression and function of P2X3 and TRPV1 receptors [18,39]. Hence, the present study was initiated to characterize BNP and NPR-A expression in the mouse TG and to examine whether the BNP/NPR-A system may modulate TRPV1 and P2X3 nociceptor activity.

## Results

### BNP and NPR-A are expressed *in vivo* in adult mouse TG

**Figure 1:**
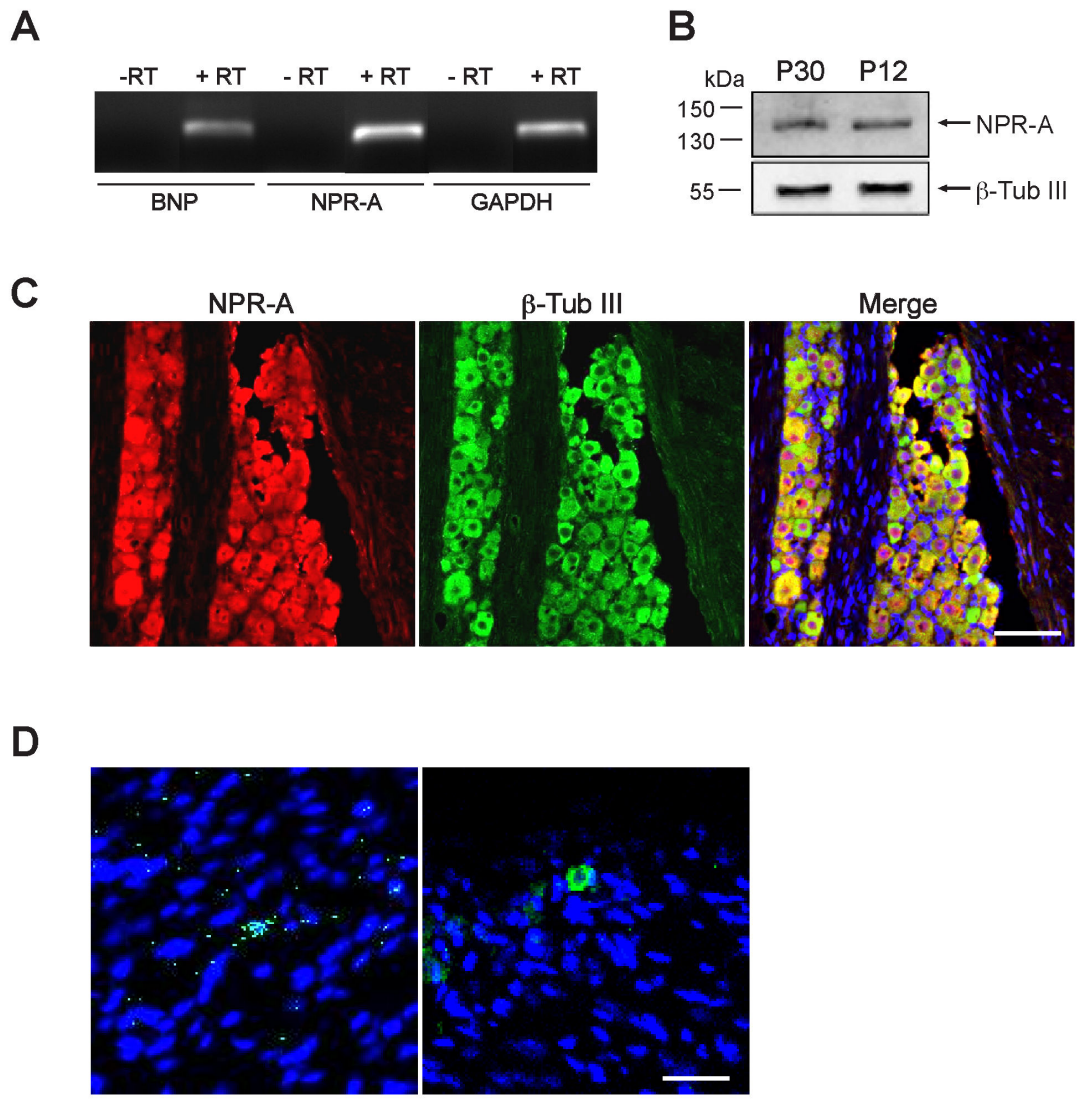
Expression of BNP and NPR-A in adult mouse TG *in vivo*. A, PCR products (amplified with 40 cycles) identified by ethidium bromide staining show RT-PCR amplification of BNP and NPR-A mRNA (+RT), as well as of the housekeeping gene GAPDH. Negative control reactions for retrotranscription (-RT) do not show any bands. B, Chemiluminescence image of Western blot showing protein expression of NPR-A in TG from P30 and P12 mice. β-Tubulin III was used as a loading control. C, Example of confocal microscopy images showing the widespread colocalization of immunostaining for NPR-A (red) and β-tubulin III (green). Cell nuclei are visualized with DAPI staining (blue). Scale bar, 100 µm. D, Representative confocal images of longitudinal sections of adult TG. Immunohistochemistry reveals BNP-immunoreactive granules around few nuclei (DAPI). Among these cells, most possess non-neuronal morphology (left panel); neuron-like cells are rarely immunostained (right panel). Scale bar, 30 µm.

Gene expression of BNP and its receptor NPR-A investigated by RT-PCR indicated weakly positive BNP and strongly positive NPR-A bands of the expected size (199 bp and 200 bp respectively; Figure 1 A). We next examined with Western blot analysis if NPR-A protein was synthesized. The NPR-A antibody recognized a single band of the expected size (approximately 140 kDa) in mouse TG homogenates, corresponding to the NPR-A protein. Conversely, BNP expression was below detection. Thus, BNP expression was evaluated by ELISA assay. Mouse BNP concentrations in tissue extracts from entire TG and in the serum were 1.12 ± 0.34 pg/mg protein and 185.2 ± 47 pg/ml, respectively (n=6 mice). Western blot analysis confirmed that NPR-A receptor was synthesized both in adult (P30) and in younger (P12) animal TG (Figure 1 B). Thus, the properties of this peptide could be investigated with younger tissues as well, a convenient preparation for primary culture studies.

The distribution of NPR-A and BNP in mouse TG was further examined by immunohistochemistry. We found that NPR-A was expressed in most neurons, as shown by co-localization with β-tubulin III (Figure 1 C). NPR-A immunoreactivity could not be detected in other cell types. Further observations (Figure S1 A) confirmed that the NPR-A staining appeared colocalized with TRPV1 and P2X3 immunopositive neurons. Unlike the data concerning its receptor, BNP immunopositive cells were few and scattered elements, in which the peptide staining appeared as small perinuclear granules (0.9 ± 0.4% of total ganglion cells, n=3) (Figure 1 D). The few BNP-immunoreactive cells seldom showed neuron-like morphology. 

### Characterization of BNP and NPR-A in TG primary cultures

**Figure 2:**
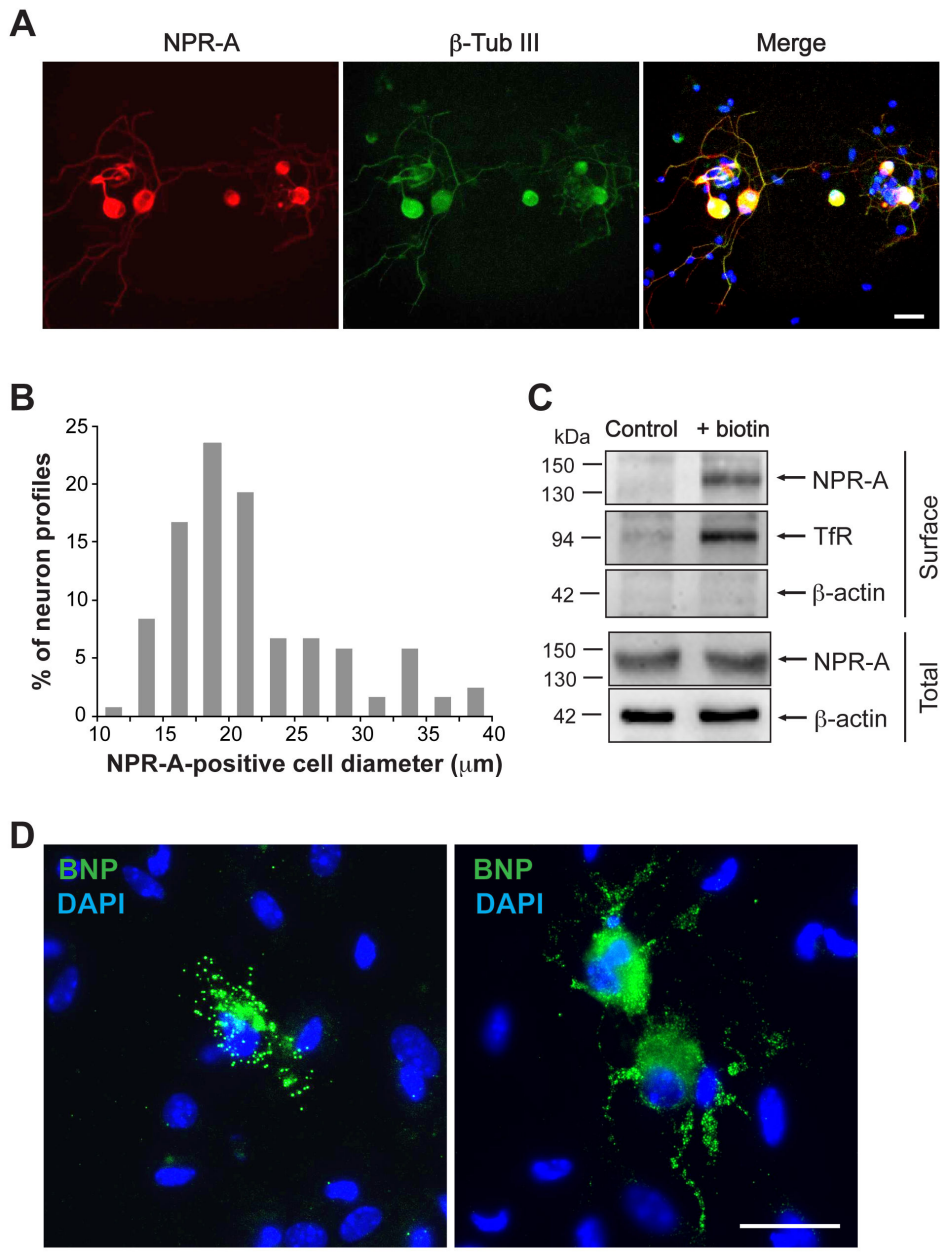
BNP and NPR-A are expressed in mouse TG cultures. A, Immunocytochemical analysis of NPR-A (red) and β-tubulin (green) in mouse TG culture. Nuclei are visualized with DAPI (blue). Merge image (right) indicates extensive co-staining. Scale bar, 30 µm. B, Somatic size distribution of NPR-A-positive TG neurons. Data represent 119 NPR-A-positive neurons from 4 independent experiments. C, Example of Western immunoblotting (representative of three experiments) showing the surface (obtained after membrane biotinylation) and total expression of NPR-A in TG culture. β-Actin was used as loadingcontrol of the total extract. TfR is the marker for correct biotinylation. D, Immunostaining for BNP (green) is mostly restricted to few cells with non-neuronal morphology that display perinuclear immunoreactive granules (left panel). More rarely, some neuron-like cells are also stained for BNP with granules extending along their processes (right panel). Nuclei are visualized with DAPI staining (blue). Scale bar, 30 µm.

In order to examine the characteristics of BNP effects on TG cells, we used TG primary cultures as previously reported [18,39]. Consistent with our results from tissue sections, double immunostaining for NPR-A and the neuronal marker β-tubulin III showed that *in vitro* the receptor was expressed exclusively by neurons (Figure 2 A). Figure 2 B summarizes the distribution of NPR-A immunoreactivity in relation to the neuronal cell body size which was the highest in neurons with somatic diameter between 12 and 25 µm, consistent with the higher prevalence of small and medium sized neurons in TG [18]. Systematic cell counting indicated that the vast majority of β-tubulin III-positive cells expressed NPR-A (91.3 ± 1.1%, n=10), confirming the extensive colocalization of the two proteins already observed *in vivo*. Western blot analysis with biotinylation method validated the expression and membrane localization of NPR-A (Figure 2 C).

Immunocytochemistry revealed the scant presence of BNP-positive cells also in TG primary cultures, as they accounted for only 2.0 ± 0.2% of the total cell population in our cultures (n=12). The morphology of these cells appeared prevalently non-neuronal (Figure 2 D, left), although rare neuron-like cells were observed as well (Figure 2 D, right). We also estimated (with ELISA assay) the concentration of BNP in primary cultures and in their medium that was found to be 1.1±0.4 pg/mg and 4.9±2.6 ng/ml, respectively (n=8 mice).

### Functional assays of NPR-A

**Figure 3:**
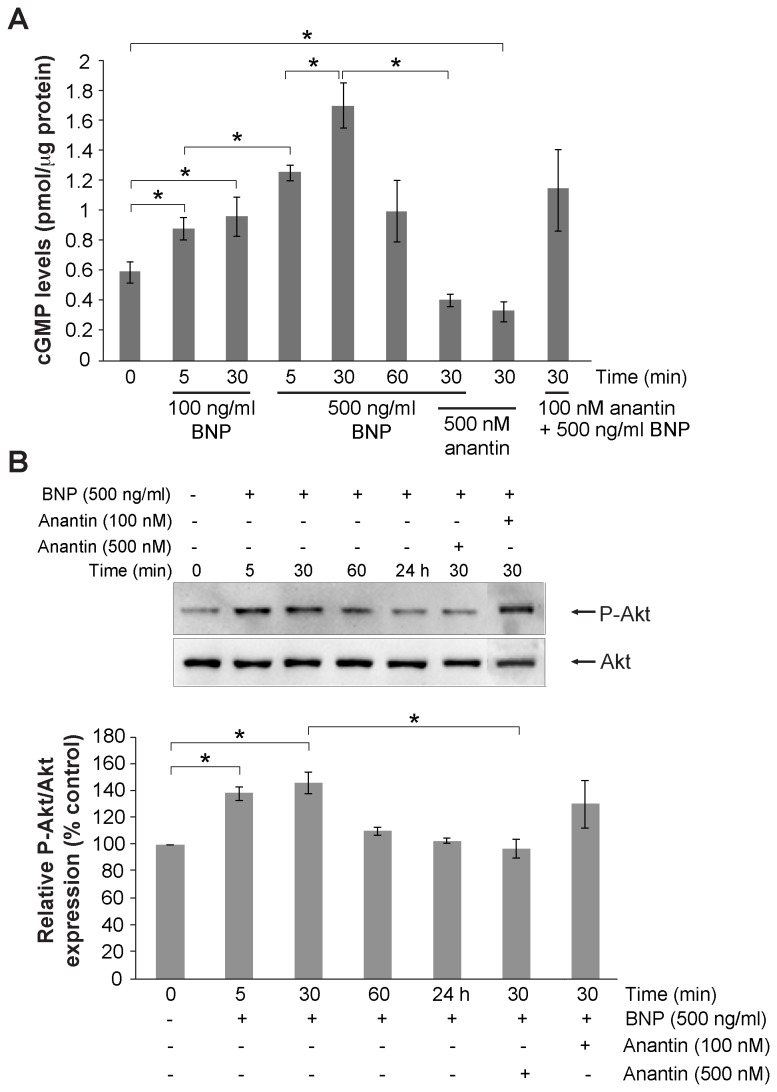
cGMP production by mouse TG cultures. A, ELISA-based quantification of intracellular cGMP in primary cultures from P12 mice. Cultures were treated with BNP (100 or 500 ng/ml for 5, 10 or 30 min) and/or anantin (100 and 500 nM, 30 min), or treated with vehicle (indicated as 0). Note large rise in cGMP that declines at 60 min; anantin alone (500 nM) decreases basal cGMP. Data were normalized to the total protein content in each sample and are presented as mean value ± SEM (n=3). *p≤0.05. B (top), example of western blot of Akt phosphorylation induced by BNP (500 ng/ml) applied alone or together with anantin (100 and 500 nM) for various times as indicated. Bottom histograms quantify these data from at least 3 experiments, demonstrating early and transient increment in P-Akt expression.

We sought to understand whether the large expression of membrane bound NPR-A was actually indicative of a functional receptor system. To this end, since activation of NPR-A by ANP and BNP has been shown to increase intracellular cGMP production [40], we investigated if application of BNP to mouse TG cultures could increase intracellular cGMP production, as determined by ELISA. Figure 3 A shows that application of 100 or 500 ng/ml BNP (compared to the vehicle control treatment) increase the mean cGMP levels in a concentration- and time-related fashion. In fact, the cGMP rise was already detectable after 5 min and reached its peak after 30 min with a late (1 h) decline. The greatest increment was observed after 30 min application of 500 ng/ml BNP. Even 100 ng/ml BNP was sufficient to activate the NPR-A mediated pathways by significantly increasing cGMP by almost 2-fold compared to basal levels. This effect was prevented by the application of 500 nM anantin, a specific antagonist of NPR-A [41,42], that completely inhibited the elevation of cGMP levels. A lower anantin concentration (100 nM) failed to produce significant antagonism of BNP-elicited rise in cGMP (Figure 3 A). It is noteworthy that anantin (500 nM) per se significantly decreased the basal level of cGMP.

One important intracellular effect of NPR-A activation is phosphorylation of Akt that occurs downstream of cGMP synthesis [43]. We tested whether this phenomenon could be also observed in TG cultures. Figure 3 B shows that after 10 or 30 min application of 500 ng/ml BNP, there was a significant increase in Akt phosphorylation, an effect fully blocked by 500 nM anantin, yet insensitive to 100 nM antagonist concentration. It was of interest that the increment in Akt phosphorylation was not sustained as it declined at 30 min and disappeared 24 h later (Figure 3 B).

These experiments, thus, demonstrated that functional NPR-A receptors were found in TG cultures despite the poor expression of BNP. We wondered if activation of these receptors could be translated into changes in the activity of ligand-gated neuronal channels considered to be important transducers of nociceptive signals. For this purpose, we studied how BNP application could affect electrophysiological responses mediated by P2X3 or TRPV1 channels.

### Effect of BNP on P2X3 and TRPV1 receptors

Patch clamp experiments were run to test whether short or long term application of 100 ng/ml BNP could change responses evoked by α,β-meATP (10 µM) or capsaicin (1 µM) to activate P2X3 or TRPV1 receptors, respectively [18]. As a reference, we also tested the inward, Cl^-^ mediated currents generated by GABA (10 µM) via activation of GABA_A_ receptors [44,45]. Only one concentration of BNP (100 ng/ml) was used for electrophysiological experiments, since we had shown it to be sufficient to activate NPR-A-mediated pathways and to increase significantly cGMP levels (Figure 3 A); the same BNP concentration was also used in previous studies, showing its effectiveness on DRG neurons [25].

**Figure 4:**
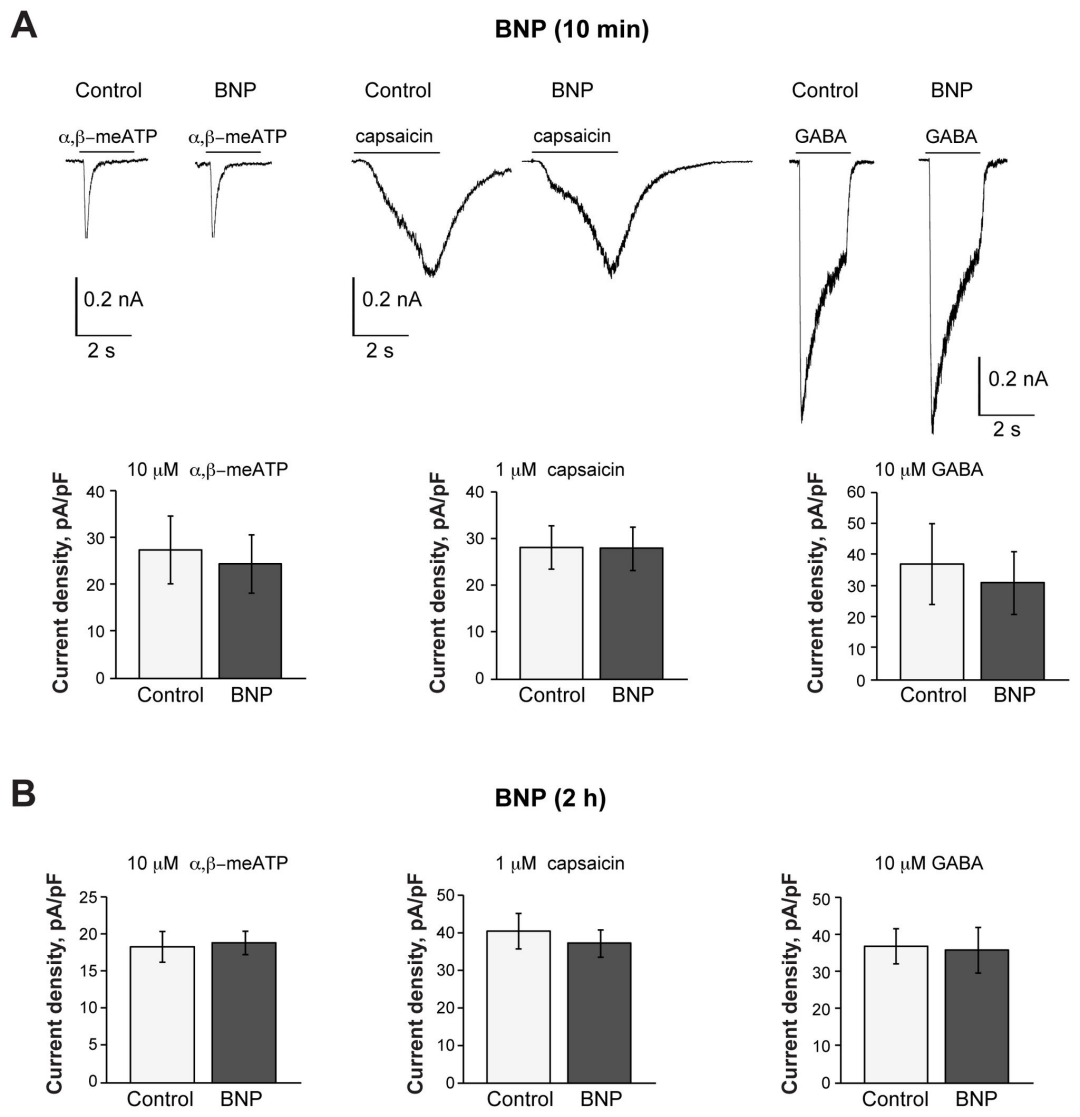
Ten min or 2 h BNP application does not affect capsaicin-, α,β-meATP- or GABA-mediated responses. A, Representative traces of currents induced by pulse application of α,β-meATP (10 µM, 2 s), capsaicin (1 µM, 3 s), or GABA (10 µM, 2 s) to TG neurons in control conditions or after application of BNP (100 ng/ml, 10 min) to the same cells. Histograms show average current density values of P2X3, TRPV1 or GABA-mediated responses (n=12, 12, 11, respectively). B, Histograms show average current density values of P2X3, TRPV1 or GABA-mediated responses in control conditions (n=52, 73, 123, respectively) or after 2 h application of 100 ng/ml BNP (n=42, 53, 119).

**Figure 5:**
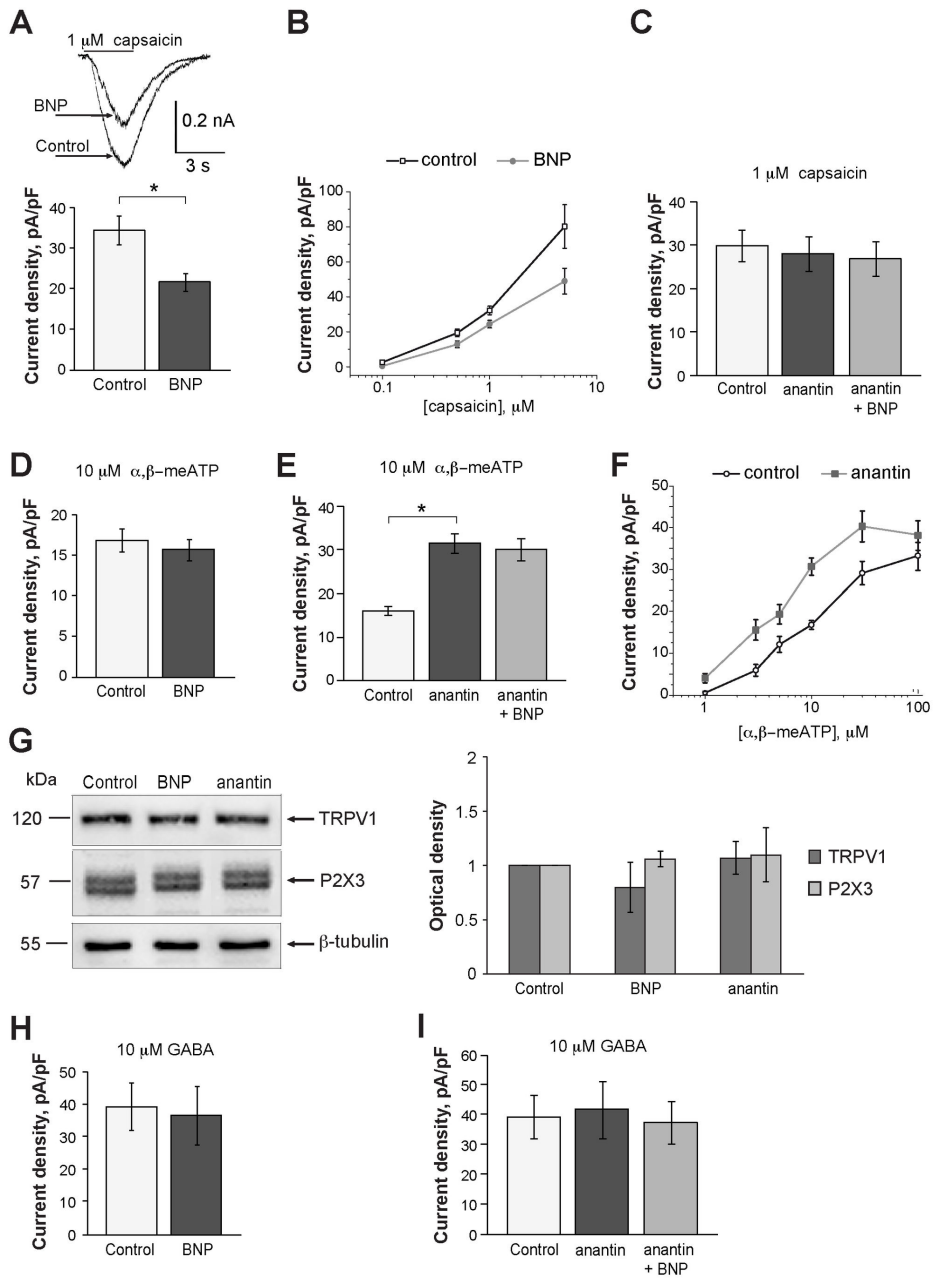
Changes in current responses produced by capsaicin, α,β-meATP or GABA following 24 h BNP application. A, Histograms show average current density values of TRPV1-mediated responses induced by 1 µM capsaicin in TG neurons under control conditions or after 24 h application of BNP (100 ng/ml); n=151 and 109 for control and after application of BNP, respectively; *p= 0.00014. Inset shows an example of superimposed currents of one neuron in control and after BNP treatment. B, Dose-response curves for capsaicin mediated currents in control and after 24 h BNP treatment (100 ng/ml); *p<0.035; n≥ 15 for each agonist concentration. C, Histograms summarize average current density values of the responses to 1 µM capsaicin in control conditions, after 24 h application of 500 nM anantin, or anantin plus 100 ng/ml BNP (n=53, 50, 43, respectively). D, E, Histograms show average current density values of P2X3-mediated currents in control conditions and after 24 h application of BNP (100 ng/ml); n=74 and 76, respectively. Anantin alone significantly increases these values compared to control, an effect not reversed by co-application with BNP (n=69, 63, 64, respectively); *p<0.0001. F, Dose-response curves for α,β-meATP mediated currents in control and after 24 h exposure to 500 nM anantin ; *p<0.035; n≥14 for each agonist concentration. G, Western blot analysis of TRPV1 or P2X3 receptor expression 24 h after applying BNP (100 ng/ml) or anantin (500 nM); data are from 3 experiments. H, I, Histograms show no change in GABA-mediated responses after 24 h application of BNP (100 ng/ml; n=25 and 27, respectively). Likewise, there is no change after application of anantin alone, or with BNP (n=73, 73, or 86, respectively).

Figure 4 A shows that continuous superfusion with BNP (100 ng/ml for 10 min) did not change sample responses to these three ligands. Furthermore, BNP did not change cell input resistance (528±50 MΩ in control versus 552±57 MΩ in the presence of BNP; n=28) or baseline current (84±15 versus 79±11 pA before and after BNP, respectively; n= 28). Preincubating cultures for 2 h with 100 ng/ml BNP also failed to alter the responses mediated by P2X3, GABA_A_ or TRPV1 receptors (Figure 4 B). Nonetheless, 24 h application of 100 ng/ml BNP significantly and selectively depressed the currents evoked by 1 µM capsaicin (Figure 5 A, see arrows). This effect was also observed for other capsaicin concentrations (0.1 µM, 0.5 and 5 µM; see Figure 5 B) as the dose/response plot was shifted downwards after chronic application of BNP. 

We next explored whether this slow action by BNP could be attributed to the consequence of NPR-A receptor activation followed by a rather slow series of events culminating in TRPV1 channel modulation. Should this hypothesis be correct, the effects of BNP ought to be suppressed by the selective antagonist of NPR-A anantin [32,41,42]. Thus, we first validated the use of anantin, that, when applied alone at 500 nM (to ensure a complete blockage of NPR-A; Figure 3 A, B), did not change control responses to capsaicin α,β-meATP. Figure S1 B shows that preapplication of anantin (500 nM; 10 min) failed to change P2X3 receptor responses. Likewise, responses evoked by capsaicin (1 µM) were 11.9±4.3 pA/pF (n=6), a value not significantly different from the one observed after anantin (9.5±5.1 pA/pF, n=4), suggesting no direct channel modulation by anantin.

Figure 5 C shows that the inhibitory effect on TRPV1 receptors seen after 24 h application of BNP was fully prevented by concomitant administration of 500 nM anantin (that per se had no effect on capsaicin-induced responses even when chronically applied). No significant change in P2X3 receptor-mediated currents was present after 24 h BNP application (Figure 5 D). It was, however, interesting to observe that anantin (500 nM; 24 h) per se enhanced responses to α,β-meATP (10 µM), that were almost twice (196 %) their controls (Figure 5 E). This effect was detected at various agonist concentrations as the α,β-meATP dose-response curve was shifted leftwards without significant change in the maximum response amplitude (Figure 5 F). When anantin plus BNP were continuously co-applied for 24 h, the potentiation of the P2X3 receptor currents persisted (Figure 5 E). Figure 5 G shows that 24 h application of BNP (100 ng/ml) or anantin (500 nM) did not change the expression level of either TRPV1 or P2X3 receptors. Finally, we did not find any change in GABA-mediated currents after applying BNP, or anantin, or their combination (Figure 5 H, I).

Although short-term BNP and/or anantin application was sufficient to produce fast changes in cGMP levels or Akt phosphorylation, modulation of TRPV1 or P2X3 currents appeared only after 24 h of BNP and/or anantin exposure. Thus, we wondered if even a short BNP or anantin treatment could trigger a very delayed receptor modulation. For this, we applied BNP (100 ng/ml) or anantin (500 nM) for 1 h only and, after overnight wash, patch-clamping was performed. With this protocol, the effects of BNP and anantin on TRPV1 or P2X3 currents were no longer observed (Figure S2).

## Discussion

The principal result of our study is the novel demonstration of widespread expression of functional NPR-A receptors in mouse TG neurons (against a modest expression of BNP), and their ability to modulate TRPV1 and P2X3 receptor activity with a characteristically delayed timecourse. Thus, these data outline a new downregulation by the BNP system of the two major nociceptive signal transducers [1-5] of TG sensory neurons.

The influence of the natriuretic peptide system on nociception has only recently come to light. A study by Zhang and colleagues [25] has shown, in control conditions, high levels of BNP and low expression of NPR-A in the rat DRG. In our experiments with *in vivo* and *in vitro* mouse TG tissue, we found a different phenomenon, namely low BNP presence and almost universal neuronal expression of NPR-A, that, with biotinylation experiments, was demonstrated to be localized at membrane level. 

The cellular distribution of BNP and NPR-A was well preserved *in vitro*, indicating that TG cultures could represent a suitable model to explore the BNP mode of action. It is noteworthy that, while the NPR-A expression was exclusively neuronal and included virtually all somatic sizes, the expression of BNP could be detected in a few non-neuronal cells, alluding to a role of this peptide in the growing family of neuropeptides involved in neuron/non-neuronal cell crosstalk at ganglion level [19,20]. The scant occurrence of BNP immunopositive cells could not be attributed to an artifact of the histological procedure as lack of signal was noted even with freshly frozen sections of the trigeminal ganglion. Some neuropeptides, like vasoactive intestinal polypeptide, galanin and NPY, are normally expressed at low or undetectable levels in sensory neurons and a specific stimulus is required for these peptides to be upregulated [46]. In keeping with this view, the concentration of BNP in ganglia in situ or in primary cultures was low, yet clearly detectable even in the extracellular medium of the primary cultures where it is expected to be concentrated by the lack of continuous blood circulation. It seems feasible that certain stimuli (yet to be identified) might raise BNP levels to become more functionally relevant for modulation of ganglion neurons.

The application of exogenous BNP rapidly evoked a large increase in cGMP level, a canonical effector of BNP [28], that peaked at about 30 min and then declined. This phenomenon was fully inhibited by the selective NPR-A antagonist anantin. Downstream of NPR-A activation and cGMP production is phosphorylation of Akt [40], an intracellular kinase targeting multiple pathways in cell signalling [47]. In accordance with this notion, BNP significantly enhanced Akt phosphorylation, an effect of transient nature (like the cGMP increment) as it was lost after 1 h. 

The relatively quick onset of biochemical effects of BNP and the widespread neuronal expression of its receptor might have suggested an early change in the activity of the receptors like TRPV1 and P2X3 on nociceptors. Nevertheless, we found no significant change in neuronal currents induced by capsaicin, α,β-meATP, or GABA when BNP was applied for up to 2 h. Likewise, in the presence of BNP, there was no change in neuronal input resistance or baseline current, implying that the peptide produced neither a rapid alteration in background conductances regulating neuronal excitability nor a direct change in membrane permeability. We suggest that the relatively fast onset of biochemical effects evoked by BNP against lack of early electrophysiological action indicates a complex intracellular signalling system that triggers comparatively slow modifications in neuronal activity. This phenomenon would be compatible with a role of BNP in controlling chronic rather than acute pain. It is worth mentioning that, in rat DRG neurons, BNP rapidly enhances the activation of voltage-dependent potassium channels only when extracellular glutamate is persistently increased [25]. 

Despite the waning of BNP-induced rise of cGMP level and Akt phosphorylation following prolonged exposure, 24 h application of the peptide significantly depressed the currents evoked by capsaicin and shifted downwards the dose/response plot, suggesting systematic inhibition of TRPV1 receptor activity. This effect was prevented by anantin that per se did not change TRPV1 receptor responses. The same BNP application protocol had no effect on P2X3 receptor responses (or those mediated by GABA). Nonetheless, chronic anantin administration per se significantly augmented currents induced by various concentrations of α,β-meATP, with a lateral shift of the dose/response plot that did not change its maximum. This finding suggested that there was ambient release of BNP sufficient to inhibit P2X3 receptors, a notion fully compatible with the ELISA assay of this peptide in culture. This suggestion is also supported by the decreased cGMP level observed in the presence of anantin alone. When the NPR-A receptors were blocked by anantin (500 nM) with consequent facilitation of P2X3 receptor activity, co-applied BNP could not reverse this phenomenon. It is noteworthy that short application of anantin (or BNP) per se did not rapidly alter currents evoked by capsaicin or α,β-meATP. Furthermore, testing neurons 24 h after a short application of anantin or BNP failed to detect any receptor modulation. Overall, these findings are consistent with the view that modulation of the responses to capsaicin or α,β-meATP was a rather delayed consequence of NPR-A receptor occupation rather than a direct interaction of anantin with TRPV1 or P2X3 channels. The observed phenomenon that a NPR-A blocker could slowly enhance P2X3 receptor activity is reminiscent of a well-known process formerly described for TRPV1 receptors that are constitutively inhibited by phospholipids such as PIP2: when PIP2 hydrolysis is suppressed, TRPV1 function is slowly augmented [48].

Although the present report did not explore the molecular mechanisms linking NPR-A activation to TRPV1 and P2X3 receptor changes, a few clues might emerge by comparing the present data with former studies of sensory ganglion modulators. While further investigations are necessary to identify the precise reason whereby the modulation by BNP of TRPV1 and P2X3 receptors occurred so late, it is worth noting that, on trigeminal ganglia, modulation of P2X3 receptors by other peptides such as CGRP [20,39], bradykinin [48,49], or NGF deprivation [50] is typically slow in onset and persistent as multiple intracellular cascades are believed to be involved. Likewise, modulation of TRPV1 receptor function by growth factors (NGF, GDNF; [51]) or the cytokine TNF-α is also slow [52]. Since after 24 h exposure to BNP or anantin there was no change in the expression of TRPV1 or P2X3 protein in ganglion cell lysates, a possible target for BNP modulation is hypothesised to be the turnover process of these receptors [53,54] which might have shifted the balance between receptor compartments at membrane and cytosol level with consequent reduction in membrane current responses.

It is also emphasized that the strong expression of NPR-A by TG neurons (despite the poor presence of BNP) would make the TG particularly sensitive to circulating BNP released by a variety of peripheral tissues under physiological and pathological conditions [28]. Hence, the NPR-A system might represent a slow regulator of sensory excitability.

While in vivo experiments using trigeminal pain conditions will be necessary to establish the functional outcome of NPR-A activity, our data suggest that BNP/NPR-A signaling could have antinociceptive functions. Conversely, NPR-C receptors are present in DRG (and colocalized with TRPV1 channels) and are activated by CNP to enhance thermal hyperalgesia in a protein G βγ- and PKC-dependent fashion [24]. These results indicate the coexistence of functional NPRs in DRG that exhibit contrasting effects in pain transduction. 

Trigeminal sensory neurons constitute the peripheral component of the so-called trigeminovascular system, an important pain complex thought to play a central role in primary headache syndromes, such as migraine [55,56]. Several neuropeptides contribute to the development of migraine pain: for example, CGRP increases the expression and the membrane targeting of P2X3 receptors [19,20,39], and bradykinin upregulates TRPV1 channels [57]. These mediators have the common ability to alter both neuronal and vascular function, a property shared by natriuretic peptides, which modulate nociceptive transmission at DRG level [24,25]. Since migraine patients display higher proBNP serum levels compared to healthy subjects [38], these data together with the present findings indicate the desirability for future studies to address the potential involvement of the natriuretic peptide system in migraine pathophysiology.

## Methods

All experiments involving the use of mice and the procedures followed therein were approved by the Scuola Internazionale Superiore di Studi Avanzati (SISSA) ethics committee and are in accordance with the European Union guidelines. Animals were maintained in accordance with the guidelines of the Italian Animal Welfare Act.

### Preparations of mouse trigeminal ganglia and immunostaining

Primary cultures of P12 mouse trigeminal ganglia were prepared as described previously [18,39] and used 24 h after plating. 

For immunohistochemistry, mice were deeply anesthetized with intraperitoneal injection of urethane (0.3 ml of 1 g/ml; Sigma, Milan, Italy) and perfused transcardially with phosphate buffer solution followed by 4% paraformaldehyde. Trigeminal ganglia were removed, postfixed for 1 h at room temperature and cryoprotected overnight in 30% sucrose at 4°C. Each immunohistochemistry experiment was performed on an average of 5 cryostat-cut serial longitudinal slices (14 µm-thick) sampled every ~70 µm, and thus covering the entire ganglion. Samples were incubated in a blocking solution containing 5% bovine serum albumin, 1% fetal bovine serum and 0.1% Triton X-100 in phosphate saline buffer for 2 h at room temperature, and immunostained with primary (for 16 h at 4°C) and secondary antibodies (2 h at room temperature). Antibodies against NPR-A (1:1000; Abcam, Cambridge, UK), BNP (1:100; Phoenix Pharmaceuticals, Burlingame, CA, USA) and β-tubulin III (1:1000; Sigma) were used. The specificity of the NPR-A antibody used in this study has been previously validated [25]. Secondary antibodies conjugated with Alexa Fluor-488 or Alexa Fluor-594 were purchased from Invitrogen (1:500; Milan, Italy). Nuclei were counterstained with DAPI (Sigma). The scant immunostaining for BNP led us to check whether this phenomenon could be attributed to a fixation artifact. Thus, we cut, with a cryostat, freshly frozen ganglia (14 µm sections) and processed them for BNP immunoreactivity using the blocking solution indicated above. Even in this case, BNP signals were absent or very rare. Double immunofluorescence experiments were performed using Zenon Technology: the anti-NPR-A antibody was labeled with Zenon Alexa-594 rabbit IgG labeling reagent as per manufacturers’ instructions (Invitrogen). Images from whole ganglion sections were visualized with Leica confocal microscope (Leica TCS SP2, Wetzlar, Germany) or a Zeiss Axioskop fluorescence microscope (Zurich, Switzerland). Similar procedures were used for fluorescence immunostaining of cultured mouse TG neurons [18,58]. MetaMorph software (Molecular Devices, Downingtown, PA, USA) was employed for data analysis.

### Western blot

Western blot analysis was performed according to the methods previously reported [58,59]. Cells were lysed in buffer solution (2% n-octyl-beta-D-glucopyranoside, contaning 1% Nonidet P-40, 10 mM Tris pH 7.5, 150 mM NaCl plus protease inhibitors mixture; Complete, Roche Applied Science, Basel, Switzerland) and immunoblotted with rabbit anti-NPR-A (1:1000, Abcam), anti-β-tubulin III (1:2000; Sigma) or anti-β-actin (1:3000, Sigma) antibodies. For Akt phosphorylation assays, cells were lysed in phosphate buffer solution containing 0.1% SDS, 1% sodium deoxycholate, and 1% triton X-100, and immunoblotted with anti-phospho-Akt Ser473 (1:1000; Cell Signaling, Danvers, MA, USA), anti-Akt (1:1000; Cell Signaling) or anti-β-actin-HRP (1:3000; Sigma). Signals were detected with the enhanced chemiluminescence light system ECL (Amersham Biosciences, Piscataway, NJ, USA) and recorded by the digital imaging system Alliance 4.7 (UVITEC, Cambridge, UK). Quantification of the optical density of the bands was performed with ImageJ software plug-in (Rasband, W.S., ImageJ, US National Institutes of Health, Bethesda, MD, USA, http://imagej.nih.gov/ij/, 1997-2012).

### Biotinylation of surface expressed receptors

For biotinylation experiments, cells were incubated with 1 mg/ml EZ-Sulfo-NHS-LC-biotin (Pierce, Rockford, IL, USA) as previously described [39,60]. Pull-down of biotinylated proteins was obtained with Streptavidin agarose resin (Pierce) for 2 h at 4°C according to the manufacturer’s instructions. Samples were processed for Western immunoblotting using antibodies against the NPR-A receptor (1:1000). Biotinylation experiments resulted to be free of intracellular protein contaminants (as shown by lack of signal for β-actin). Positive control for biotinylation assay was obtained by checking the surface expression of the transferrin receptor detected with an antibody purchased from Santa Cruz Biotechnology (1:1000, Heidelberg, Germany). For control of correct gel loading, the expression of β-actin in the intracellular fraction was checked. 

### RNA isolation, reverse transcription and RT-PCR

Total RNA was extracted from mouse intact TG tissue samples using Trizol reagent (Invitrogen) according to the manufacturer’s instruction. All RNA samples were subjected to DNase I treatment (Ambion, Monza, Italy). A total of 1 μg of RNA was subjected to retrotranscription using iScript cDNA synthesis kit (BioRad, Hercules, CA, USA) and RT–PCR was carried out using SYBR green fluorescence dye (2× iQ5 SYBR Green supermix, BioRad) as described previously [61]. Specific primer sets for NPR-A [62], BNP [63], GAPDH [64] and β-actin [65] were previously described. 

### Assay for intracellular cGMP production in TG cultures

Quantification of BNP-induced intracellular cGMP production in mouse TG cultures was performed using a direct ELISA kit (MBL International Corporation, Woburn, MA, USA) following the instructions of the manufacturer. Briefly, cultures were lysed in 0.1 M HCl and subjected to determination of intracellular cGMP concentration. The protein concentrations in cell lysates were determined by the BCA method (Sigma). Intracellular cGMP concentrations were expressed as pmol of cGMP per µg of protein. Three independent experimental replicates were generated from three different batches of mouse TG cultures.

### Serum sampling and ELISA assay for BNP

Blood was sampled from the retro-orbital sinus of mice, immediately transferred to chilled microtubes on ice for 30 min and centrifuged at 10,000 *g* at 4°C for 10 min. Culture medium was collected from TG cultures 24 h after plating and centrifuged at 10,000 *g* at 4°C for 10 min. From the same cultures, cells were lysed in 0.1 M HCl and centrifuged at 10,000 *g* at 4°C for 10 min. Protein concentrations in cell lysates were determined by the BCA method (Sigma). Tissue and medium samples were immediately used for ELISA assay to determine BNP concentrations following the instructions of the manufacturer (Abnova, Hidelberg, Germany). All samples were run in triplicate and values averaged.

### Electrophysiology and data analysis

Electrophysiology experiments were performed according to the methods previously reported [17,18]. Briefly, after 24 h in culture, TG neurons were superfused continuously (3 mL/min) with physiological solution containing (in mM): 152 NaCl, 5 KCl, 1 MgCl_2_, 2 CaCl_2_, 10 glucose, and 10 HEPES (pH adjusted to 7.4 with NaOH). Cells were patch-clamped in the whole-cell configuration using pipettes with a resistance of 3-4 Mʊ when filled with the following solution (in mM): 140 KCl, 0.5 CaCl_2_, 2 MgCl_2_, 2 Mg_2_ATP_3_, 2 GTP, 10 HEPES, and 10 EGTA (pH adjusted to 7.2 with KOH). Recordings were performed from small and medium size neurons that mostly express TRPV1 and/or P2X3 receptors [17,18]. Cells were held at -65 mV; membrane currents were filtered at 1 kHz and acquired by means of a DigiData 1200 interface and pClamp 8.2 software (Molecular Devices, Sunnyvale, CA, USA). To obtain stable and reproducible P2X3 receptor currents, its synthetic agonist α,β-methylene-adenosine-5’-triphosphate (α,β-meATP; Sigma) at the concentration of 10 µM that elicits near-maximal responses [58,64,66] was applied (for 2 s) using a fast superfusion system (Rapid Solution Changer RSC-200; BioLogic Science Instruments, Claix, France). α,β-meATP at 1, 3, 5, 30 and 100 µM concentrations was additionally studied to obtain dose-response curves in control and experimental conditions. In line with previous reports [17,58], capsaicin (Sigma) was applied at the concentration of 1 µM (3 s) to evoke reproducible inward currents. Additionally concentrations 0.1, 0.5, 5 and 10 µM were included to obtain capsaicin dose-response curves under control conditions and after the treatment. GABA (10 µM) was applied with 2 s pulses [45]. Peak current amplitudes were divided by the cell slow capacitance to express responses as current density values.

For patch clamp experiments, various protocols were used to test the effect of BNP on these ligand-gated channels. In a series of experiments, BNP was continuously superfused (starting 10 min prior to the agonist pulses) at 100 ng/ml concentration while agonist responses were repeatedly tested. In other experiments, TG cultures were incubated with BNP solution (100 ng/ml) for 2 or 24 h; after washing out the peptide, cells were immediately patch-clamped and tested for their responsiveness to the agonists. In the third type of the protocol BNP was applied for 1 h and, after wash out, cells were incubated for 23 h in the usual medium before the experiment. A similar approach was employed for the NPR-A antagonist anantin (500 nM) applied either alone or together with BNP. 

### Chemicals and reagents

Purified recombinant mouse BNP was purchased from Phoenix Pharmaceuticals; the specific NPR-A antagonist anantin was from US Biologicals (Salem, MA, USA). BNP and anantin were dissolved in distilled water as the datasheet suggested. BNP was applied at the dose of 100 ng/ml, as previously performed for sensory ganglia [25], and 500 ng/ml. Anantin was applied at the dose of 100 nM and 500 nM as previously reported [67].

To inhibit NPR-A receptor activity, we used anantin since, to the best of our knowledge, it is the only specific and selective NPR-A antagonist [41–43], devoid of agonistic activities [68], unlike commercially-available antagonists which are not selective on NPR subtypes [69–72], and which may also possess agonistic activities [73].

### Statistical analysis

Data were collected from at least three independent experiments, and are expressed as mean ± standard error of the mean (SEM), where *n* indicates the number of independent experiments or the number of investigated cells, as indicated in figure legends. Statistical analysis was performed using the Student’s *t*-test or the Mann-Whitney rank sum test, after the software-directed choice of parametric or non-parametric data, respectively (Sigma Plot and Systat Software Inc., San Jose, CA, USA). A p value of ≤ 0.05 was accepted as indicative of a statistically significant difference.

## Supporting Information

Figure S1 **Co-localization of NPR-A with TRPV1 and P2X3 receptors, and lack of acute NPR-A block on P2X3 receptor responses.** A, Example of confocal microscopy images showing the colocalization of immunostaining for NPR-A (red) and TRPV1 or P2X3 (green). Cell nuclei are visualized with DAPI staining (blue). Scale bar, 30 µm. B, Representative traces of currents induced by pulse application of α,β-meATP (10 µM, 2 s) to TG neurons in control conditions or after application of anantin (500 nM, 10 min) to the same cells. Histograms show average current density values of P2X3-mediated currents (n=18).
(TIF)

Figure S2 **1 h BNP or anantin application has no effect on capsaicin-, α,β-meATP- or GABA-mediated responses recorded 23 h later.** Histograms show average current density values of P2X3, TRPV1 or GABA-mediated responses in control conditions (n=40, 58, 82, respectively) or 23 h after 1 h application of 100 ng/ml BNP (n=32, 66, 98) or 500 nM anantin (n=39, 38, 64).
(TIF)
