# Supplementary figures and images for: B-Type Natriuretic Peptide-Induced Delayed Modulation of TRPV1 and P2X3 Receptors of Mouse Trigeminal Sensory Neurons
Source: PLoS One. 2013 Nov 27;8(11):e81138. doi: 10.1371/journal.pone.0081138 (PMC3842315; doi:10.1371/journal.pone.0081138)

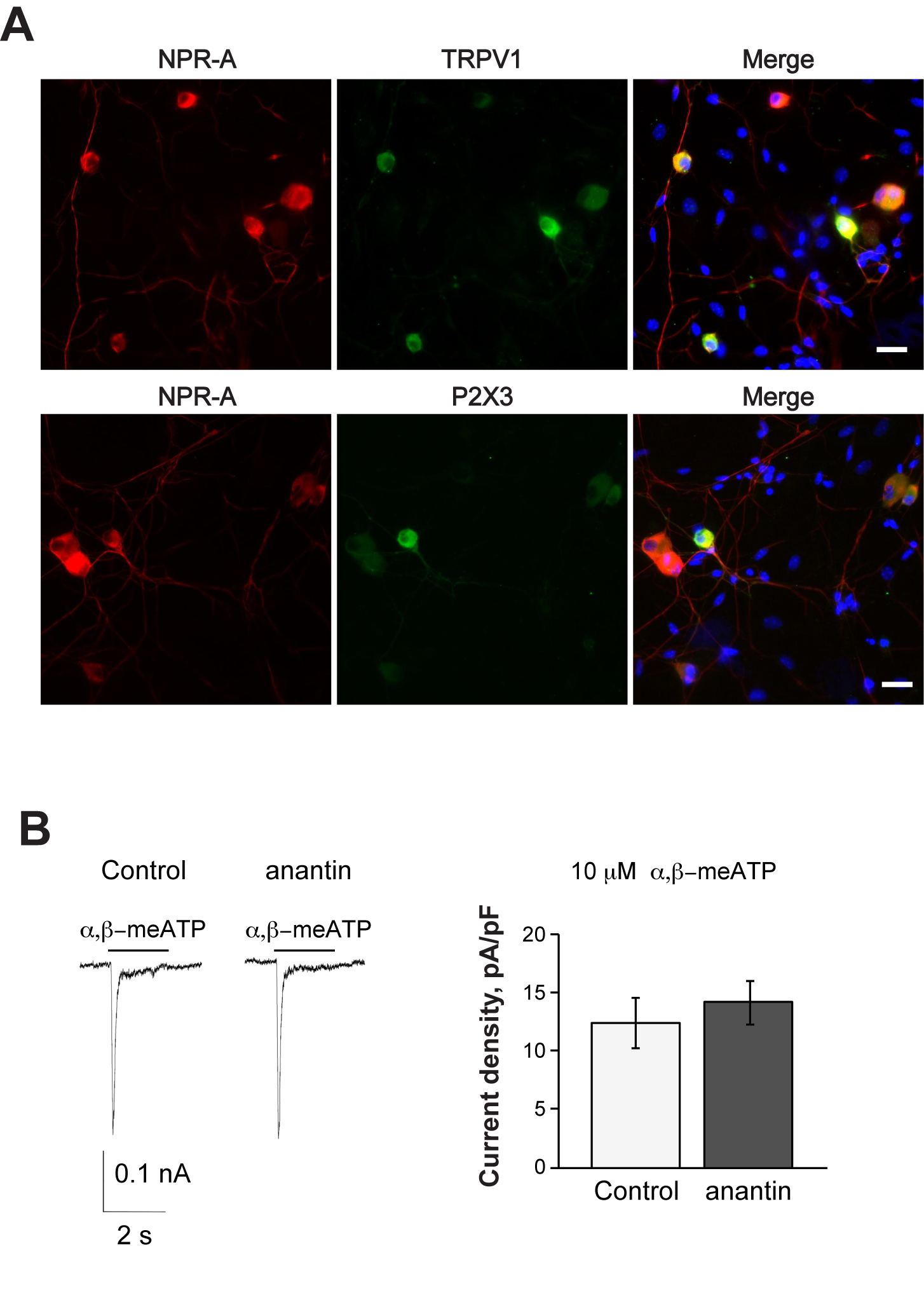

Supplement: Figure S1 — Co-localization of NPR-A with TRPV1 and P2X3 receptors, and lack of acute NPR-A block on P2X3 receptor responses. A, Example of confocal microscopy images showing the colocalization of immunostaining for NPR-A (red) and TRPV1 or P2X3 (green). Cell nuclei are visualized with DAPI staining (blue). Scale bar, 30 µm. B, Representative traces of currents induced by pulse application of α,β-meATP (10 µM, 2 s) to TG neurons in control conditions or after application of anantin (500 nM, 10 min) to the same cells. Histograms show average current density values of P2X3-mediated currents (n=18). (TIF) [file pone.0081138.s001.tif]

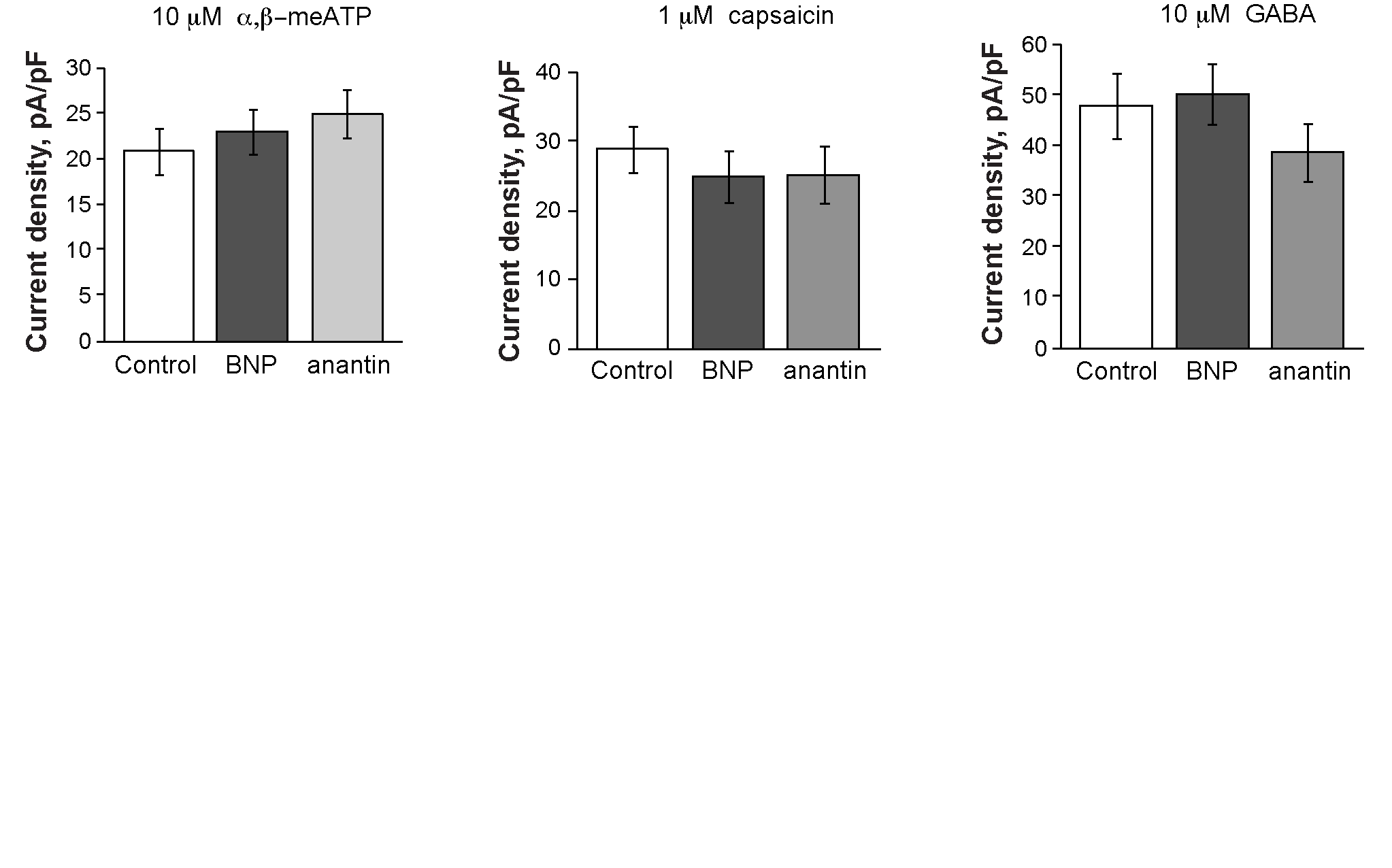

Supplement: Figure S2 — 1 h BNP or anantin application has no effect on capsaicin-, α,β-meATP- or GABA-mediated responses recorded 23 h later. Histograms show average current density values of P2X3, TRPV1 or GABA-mediated responses in control conditions (n=40, 58, 82, respectively) or 23 h after 1 h application of 100 ng/ml BNP (n=32, 66, 98) or 500 nM anantin (n=39, 38, 64). (TIF) [file pone.0081138.s002.tif]
